# Predicting Psoriatic Arthritis in Psoriasis Patients – A Swiss Registry Study

**DOI:** 10.1177/24755303231217492

**Published:** 2023-11-21

**Authors:** Mia-Louise Nielsen, Troels C. Petersen, Lara Valeska Maul, Jacob P. Thyssen, Simon F. Thomsen, Jashin J. Wu, Alexander A. Navarini, Thomas Kündig, Nikhil Yawalkar, Christoph Schlapbach, Wolf-Henning Boehncke, Curdin Conrad, Antonio Cozzio, Raphael Micheroli, Lars Erik Kristensen, Alexander Egeberg, Julia-Tatjana Maul

**Affiliations:** 1 Department of Dermatology, Bispebjerg Hospital, 4321 University of Copenhagen, Copenhagen, Denmark; 2 Niels Bohr Institute, 4321 University of Copenhagen, Copenhagen, Denmark; 3 Department of Dermatology, 30262 University Hospital Basel, Basel, Switzerland; 4 Department of Dermatology, University of Miami Miller School of Medicine, Miami, FL, USA; 5 Faculty of Medicine, University of Zürich, Zürich, Switzerland; 6 Department of Dermatology, 27243 University Hospital Zürich, Zürich, Switzerland; 7 Department of Dermatology, Inselspital, Bern University Hospital, 27252 University of Bern, Bern, Switzerland; 8 Division of Dermatology and Venereology, 27230 Geneva University Hospitals, Geneva, Switzerland; 9 Department of Dermatology, CHUV University Hospital and University of Lausanne (UNIL), Lausanne, Switzerland; 10 Department of Dermatology, Venereology and Allergy, Cantonal Hospital St. Gallen, St. Gallen, Switzerland; 11 Department of Rheumatology, 27243 University Hospital Zürich, Zürich, Switzerland; 12 The Parker Institute, Bispebjerg and Frederiksberg Hospital, 4321 University of Copenhagen, Copenhagen, Denmark; 13 Department of Clinical Medicine, 4321 University of Copenhagen, Copenhagen, Denmark

**Keywords:** classification, machine learning, predictive models, psoriasis, psoriatic arthritis, statistics, real word, registry

## Abstract

**Background:**

Psoriatic arthritis (PsA) is a prevalent comorbidity among patients with psoriasis, heavily contributing to their burden of disease, usually diagnosed several years after the diagnosis of psoriasis.

**Objectives:**

To investigate the predictability of psoriatic arthritis in patients with psoriasis and to identify important predictors.

**Methods:**

Data from the Swiss Dermatology Network on Targeted Therapies (SDNTT) involving patients treated for psoriasis were utilized. A combination of gradient-boosted decision trees and mixed models was used to classify patients based on their diagnosis of PsA or its absence. The variables with the highest predictive power were identified. Time to PsA diagnosis was visualized with the Kaplan-Meier method and the relationship between severity of psoriasis and PsA was explored through quantile regression.

**Results:**

A diagnosis of psoriatic arthritis was registered at baseline of 407 (29.5%) treatment series. 516 patients had no registration of PsA, 257 patients had PsA at inclusion, and 91 patients were diagnosed with PsA after inclusion. The model’s AUROCs was up to 73.7%, and variables with the highest discriminatory power were age, PASI, physical well-being, and severity of nail psoriasis. Among patients who developed PsA after inclusion, significantly more first treatment series were classified in the PsA-group, compared to those with no PsA registration. PASI was significantly correlated with the median burden/severity of PsA (*P* = .01).

**Conclusions:**

Distinguishing between patients with and without PsA based on clinical characteristics is feasible and even predicting future diagnoses of PsA is possible. Patients at higher risk can be identified using important predictors of PsA.

## Introduction

Psoriasis is an immune-mediated inflammatory disease manifesting in the skin but considered to be systemic in nature. Psoriatic arthritis (PsA) is one of the most common comorbidities associated with psoriasis, which further burdens the patients and impairs their life quality. Approximately 25% of patients with psoriasis receive a diagnosis of PsA, making it one of the most common comorbidities.^1,2^

The relationship between the pathogenesis of cutaneous psoriasis and PsA remains an active field of study. Dedicated research has investigated the genomic profiles of patients with psoriasis only and those who also develop PsA. Their results indicate an overlap between genomes associated with psoriasis and PsA, however, some genetic markers are found to be risk factors only for either psoriasis or PsA.^3–5^

PsA affects the joints, usually by swelling, stiffness, and pain. Without treatment, the disease can get progressively worse, posing the risk irreversible joint damage. It can severely worsen the physical well-being of the patients, preventing them from performing everyday activities.^6^

Typically, PsA is diagnosed several years after the diagnosis of psoriasis. Psoriatic arthritis is considerably underdiagnosed among patients with cutaneous psoriasis,^7,8^ which may impede proper and timely treatment and support from rheumatologists. Since psoriasis often precede symptoms of PsA, dermatologists are ideally positioned to screen patients for PsA, potentially making the initial diagnosis and facilitating early treatment.^9–11^ Although screening tools for early detection of PsA in patients with psoriasis exist, undiagnosed PsA remains an issue.^12^

Several systemic therapies used in the treatment of moderate-to-severe psoriasis are also approved for treating PsA. Hence, if a patient has both diagnoses, it may be possible to simultaneously treat both conditions. However, currently, many psoriasis patients without PsA diagnoses are undertreated, especially if their psoriasis is too mild to warrant systemic/biologic treatment.

Machine-learning techniques are used for a variety of purposes within medical research.^13^ Unsupervised methods can identify patterns in populations, while supervised methods are employed in classification and regression problems e.g., to predict treatment outcomes^14^ or to identify specific diseases.^15^

In this study, we applied supervised machine-learning algorithms and statistical analysis to examine the predictability of PsA development among patients with psoriasis and identify crucial predictors.

## Methods

### Data Sources

Utilizing data from the Swiss Dermatology Network on Targeted Therapies (SDNTT), this study investigated the predictability of PsA in patients with psoriasis. The SDNTT is a Swiss register containing sociodemographic and disease history data on patients with psoriasis treated with targeted therapies within five large university hospitals (Zürich, Basel, Bern, Lausanne, Geneva) as well as three cantonal/tertiary hospitals (Bellinzona, St. Gallen, Aarau) throughout Switzerland.

In the SDNTT, adult patients with moderate-to-severe psoriasis initiating a novel systemic therapy, either biologic or non-biologic, which they have not previously utilized, were enrolled. The registry captured real-world data, as it included patients with comorbidities and those on concomitant medications. Study visits were initially planned at baseline, at 3 months, and then at 6-month intervals for a follow-up period extending up to 10 years. The systemic treatments encompassed conventional therapies (such as methotrexate, cyclosporine, retinoids, and phototherapy), novel small molecule therapies (e.g., apremilast), and biologics (including anti-TNFα agents: infliximab, adalimumab, etanercept, and certolizumab pegol; anti-IL-12/23 agents: ustekinumab; anti-IL-17 agents: ixekizumab and secukinumab; and anti-IL-23 agents: guselkumab, tildrakizumab, and risankizumab). Treatment decisions were made according to the European Guidelines.^16,17^

Part of the data were recorded by the dermatologist during the patient’s visit, while other information was obtained subsequently based on a questionnaire filed by the patient in relation to the visit. The dermatologist recorded variables such as Psoriasis Area and Severity Index (PASI), information on PsA and other comorbidities, adverse effects, and treatment-specific data. The PsA diagnosis in our study was based on a rheumatologist's evaluation, however not strictly confined to the CASPAR criteria, whose primary intention is to create a homogeneous population for clinical trials; by adopting this approach, our goal was to authentically represent the diverse PsA patient population encountered in everyday clinical practice. The patient provided information on variables such as the Dermatology Life Quality Index (DLQI) and general physical well-being (EQ-5D).

Treatment series may begin and end in-between scheduled visits. In such cases, information, such as drug, start date, end date, and other details were often registered at the data entry for the following visit.

### Data Preparation

All therapy start dates were paired with the matching end date to aggregate the data into treatment series, where each treatment series corresponds to a patient treated with a single drug. A patient can have multiple treatment series with the same or different drugs.

Baseline information for treatment series was often available at the data entry corresponding to the visit of the start date. However, we assumed baseline information at the nearest visit within 14 days of the treatment start to account for some treatments being initiated between scheduled visits.

Information of nail psoriasis was included as a binary variable available in the registry. Additionally, the registry contained three variables indicating the number of nails affected: more than 90%, between 50% and 90%, and less than 50%. A nail score (henceforth referred to as “nail-score”) was calculated as the sum of these variables, weighted by 3, 2, and 1, respectively. The nail-score is 0 for no nail psoriasis and increases with the severity of affected nails.

### Statistical Analysis and Predictive Models

In this study, supervised learning was used to explore predictability of PsA and identify patient characteristics predicting PsA. We used a binary classification model trained on baseline data for each treatment series. The target variable was PsA at baseline and the patient characteristics, sex, age at diagnosis, age at beginning of treatment series, weight, BMI, PASI, DLQI, EQ-5D, and nail involvement of psoriasis (the binary yes/no variable and the continuous nail-score) were included in the model as features (predictors). The previous number of treatment series, and previous number of treatment series with biologics were included in one model and excluded in another to compare the results.

A combination of gradient boosted decision trees and mixed models were used. Gradient boosted decision trees often perform well on tabulated data and can learn non-linear relationships between the target variables and features. The purpose of the mixed model component of the algorithm aimed to account for the repeated measure aspect of the data arising from some patients having multiple treatment series.

To quantify the model performance, we performed a standard 10-fold-cross-validation with a 3-fold-cross-validation nested within. In the 10-fold cross validation, all treatment series were shuffled and split into 10 equal parts, with one part functioning as a test set in each iteration. To prevent the model from relying on information from future events for a given patient (which would introduce immortal-time bias), all treatment series for a patient initiated later than a treatment series for the same patient included in the test data were removed from the training data. Hyperparameters of the model were tuned within the inner loop of the cross-validation, while performance metrics (receiver operating curves (ROC) and the area under it (AUROC)) were estimated on the test set and averaged over the outer loop of the cross-validation. Hyperparameters include classification threshold and parameters related to gradient boosting.

Two models were trained separately: Model 1 did not include data on the previous number of treatment and biologics, whereas Model 2 did.

Missing values were imputed with a k-nearest neighbor approach for relevant features.

Shapley additive explanation (SHAP) values were used to explain predictions of the model, including feature importance, direction of impact of features on the predictions, as well as a more detailed relationship between model predictions and features. SHAP values measure the contribution of a feature/variable on the output of the mode (likelihood of a patient belonging in the PsA group) and are generally used to interpret and explain predictions of a classification model.

A Fisher’s exact test compared the number of first treatment series of each patient, that was classified as having PsA from the baseline for patients who were diagnosis with PsA after inclusion and patients with no registered PsA.

Quantile regression was used to investigate the relationship between the severity of psoriasis measures by PASI and the severity/burden of PsA measured by the sum of the numbers of joints that were swollen/painful as registered by a dermatologist. Baseline data was included for patients with a PsA diagnosis.

## Results

A total of 1379 treatment series were included, distributed across 864 unique patients. PsA was registered at the baseline of 407 (29.5%) treatment series. For 516 (59.7%) patients, there were no registration of PsA, whereas 257 (29.7%) patients had PsA at the time of their first treatment, and 91 (10.5%) patients were diagnosed with PsA during a subsequent visit (Table 1

**Table 1.** Characteristics for Treatment Series and Patients.

	Treatment series (Each Patient can Count Multiple times)			Patients at Baseline (Patients Only Count once – Information is at Baseline)		
	Overall	No PsA	PsA	No PsA	PsA at Beginning	PsA Developed after Inclusion
Number of treatment series or number of patients	1379	972	407	516	257	91
Sex, men, n (%)	492 (35.7)	350 (36.0)	142 (34.9)	193 (37.4)	96 (37.4)	31 (34.1)
Number of previous treatments, median (IQR)	.0 (.0-1.0)	.0 (.0-1.0)	1.0 (.0-2.0)	N/A	N/A	N/A
Number of previous biologics, median (IQR)	.0 (.0-.0)	.0 (.0-.0)	.0 (.0-1.0)	N/A	N/A	N/A
Age at diagnosis, median (IQR)	25 (16-39)	24 (17-38)	28 (15-39)	24 (17-38)	30 (18-39)	27 (14-40)
Age at prescription start, median (IQR)	46 (35-57)	45 (34-56)	49 (38-57)	43 (32-54)	48 (38-57)	46 (35-57)
Nail involvement, n (%)	694 (50.3)	461 (47.4)	233 (57.2)	289 (56.0)	165 (64.2)	52 (57.1)
Nail involvement, score, median (IQR)	2.0 (.0-10.0)	2.0 (.0-10.0)	2.0 (.0-10.0)	2.0 (.0-10.0)	3.0 (.0-10.0)	2.0 (.0-10.0)
BSA, median (IQR)	8.0 (3.4-15.0)	9.0 (4.0-15.0)	6.0 (3.0-11.4)	10.0 (5.3-19.0)	8.0 (3.7-15.4)	7.0 (3.0-13.7)
Weight, median (IQR)	80.0 (70.0-95.0)	80.0 (69.0-93.0)	82.5 (70.0-97.0)	80.0 (68.0-92.0)	81.0 (70.0-95.0)	80.0 (70.0-89.0)
BMI, median (IQR)	26.8 (23.5-31.2)	26.7 (23.4-31.0)	27.3 (23.8-31.7)	26.2 (23.2-30.4)	27.1 (23.9-31.9)	27.4 (23.8-30.4)
DLQI, median (IQR)	10.0 (4.0-16.0)	10.0 (4.0-16.0)	10.0 (4.0-15.0)	11.0 (5.0-17.0)	11.0 (6.0-17.0)	12.0 (5.5-15.5)
PASI, median (IQR)	7.2 (3.5-11.7)	7.7 (3.9-12.0)	6.4 (2.8-10.4)	8.9 (5.1-12.5)	7.8 (3.7-12.2)	7.0 (4.4-11.9)
EQ-5D (general physical well-being), median (IQR)	65.0 (45.0-80.0)	69.0 (50.0-80.0)	60.0 (40.0-80.0)	70.0 (50.0-80.0)	60.0 (40.0-75.0)	63.0 (47.5-79.5)

Abbreviations: IQR, interquartile range; BSA, body surface area; BMI, body mass index; DLQI, dermatology life quality index; PASI, psoriasis area and severity index; EQ-5D, ; N/A, not applicable, PsA, psoriatic arthritis.

).

Among treatment series where the patient did not have PsA, the median ages at diagnosis and at baseline of treatment were 24 and 45 years, respectively. For treatment series where patients were diagnosed with PsA prior to or at the time of the start of the treatment series, the corresponding ages were 28 and 49 years (Table 1).

In 57.2% of the treatment series among patients with PsA, there were nail involvement of psoriasis, while this was only the case for 47.4% of treatment series among patients without PsA (Table 1).

The median PASI was slightly lower at baseline for treatment series where the patient was diagnosed with PsA compared to treatment series where the patient did not have PsA (PASI = 6.4 compared to PASI = 7.7). The median DLQI scores were similar for both groups (median DLQI = 10). The median general physical well-being (EQ-5D) was 60.0 for treatment series of patients with PsA and 69.0 for treatment series where the patient was not diagnosed with PsA, indicating better general physical well-being among patients without PsA (Table 1).

**Figure 1.**
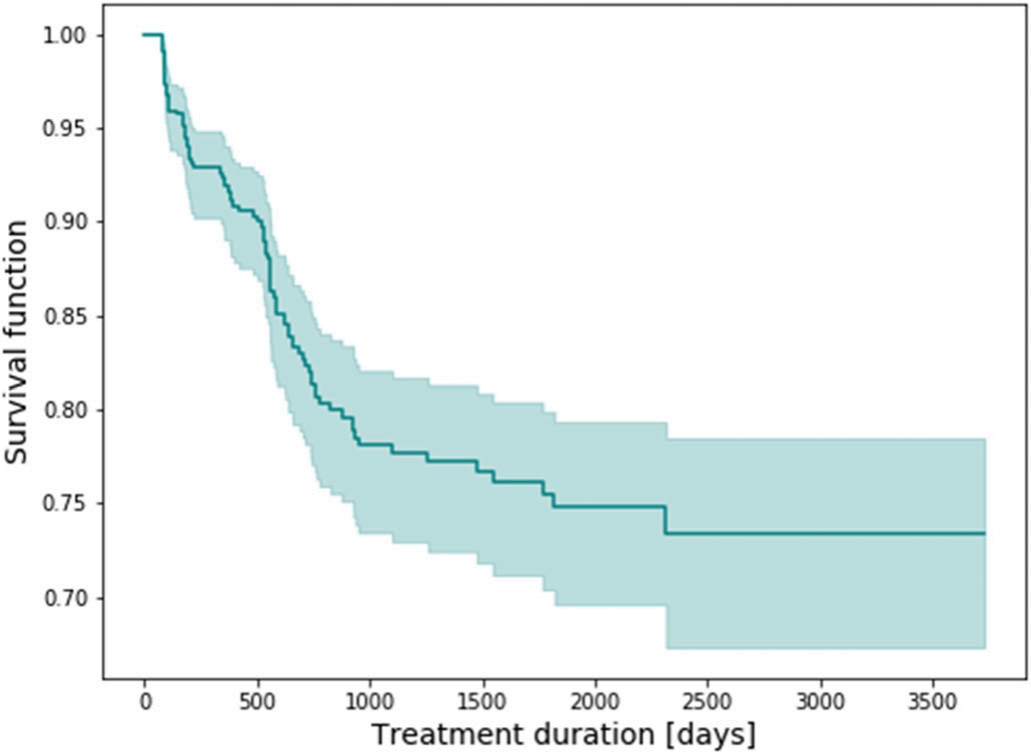
Kaplan-Meier plot showing the time to PsA diagnosis for patients with at least one follow-up visit (at least two visits in total) who did not have PsA at baseline.

The time to receiving a PsA diagnosis among patients with no PsA at baseline who had at least one follow-up visit (i.e., at least two visits in total), was estimated based on the Kaplan-Meier approach (Figure 1).

### PsA vs no PsA at Baseline – Results From a Machine-Learning Model

**Figure 2.**
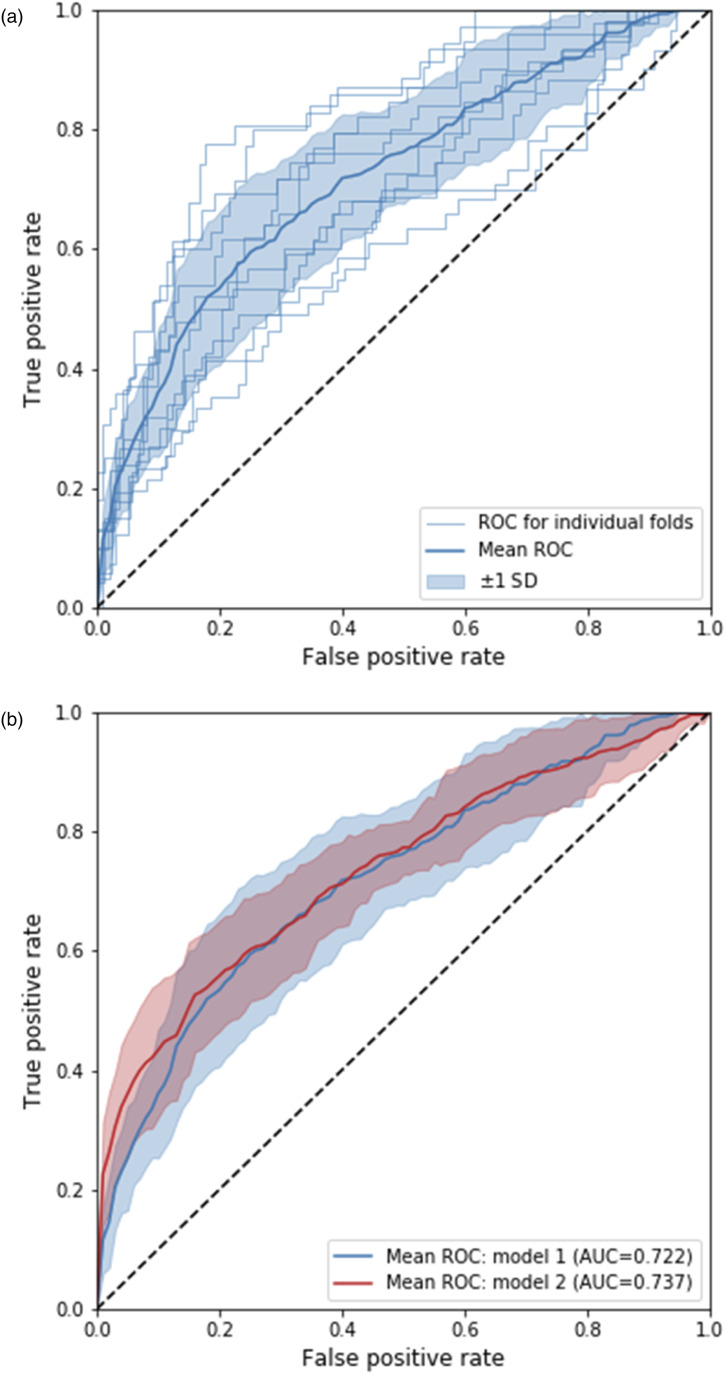
(A) Receiver operating curves for individual folds in 10-fold cross validation for model 1 (does not include information on previous number of treatments/biologics) and the average over all folds. (B) Average receiver operating curves for models 1 and 2.

The models combining gradient boosted decision trees and mixed model effects were evaluated using cross-validation, yielding AUROCs of 73.7% and 72.2% when information of the previous number of treatments/biologics was included and when it was not. The ROC was estimated and visualized for each of the 10-folds in the cross validation for model 1 (no information of number of treatment attempts) and the average ROC for both models (Figure 2).

**Figure 3.**
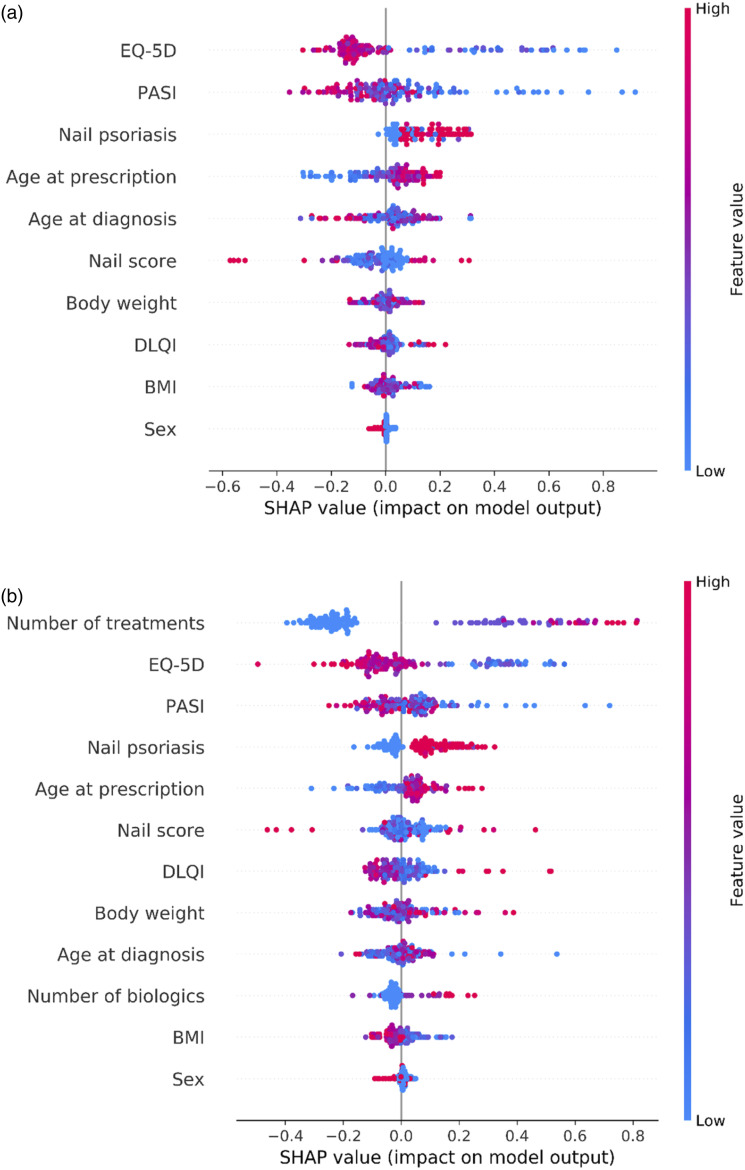
SHAP values for model 1 (A) and model 2 (B), indicating the feature importance for each variable included in the models. A higher SHAP value means that the data point (treatment series) contributed more towards a prediction of PsA.

The feature importance for both models were estimated using SHAP values (Figure 3). The colour scale represents the value of the variable (e.g., more pink symbolizing higher PASI, DLQI etc.), whereas the SHAP values are depicted along the x-axis. Each point corresponds to a single classification (a patient) in the model. SHAP values for the variables sex and the binary indicator for nail psoriasis are centered near 0, indicating no or poor discriminatory value. Some of the most important variables for model 1 were EQ-5D, PASI, and the presence of nail psoriasis. The same variables had high discriminatory value for model 2, but also the previous number of treatment series were important.

In the validation of model 1 without the previous number of treatment series as variable, there were significantly more patients who were classified as having PsA at baseline among patients who developed PsA after baseline, compared to patients who had no registration of PsA (*P* = .036). For model two, we found no difference between the number of patients who was classified as having PsA between the two groups (no registration of PsA vs PsA diagnosed during follow-up, *P* = .34).

The SHAP values for the variables with highest discriminatory power for the two models were visualized. We observed that the models captured non-linear relationships between the variables and the likelihood of a patients having PsA (Supplementary figure 1).

### Relationship Between PASI and the Burden of PsA – a Quantile Regression

**Figure 4.**
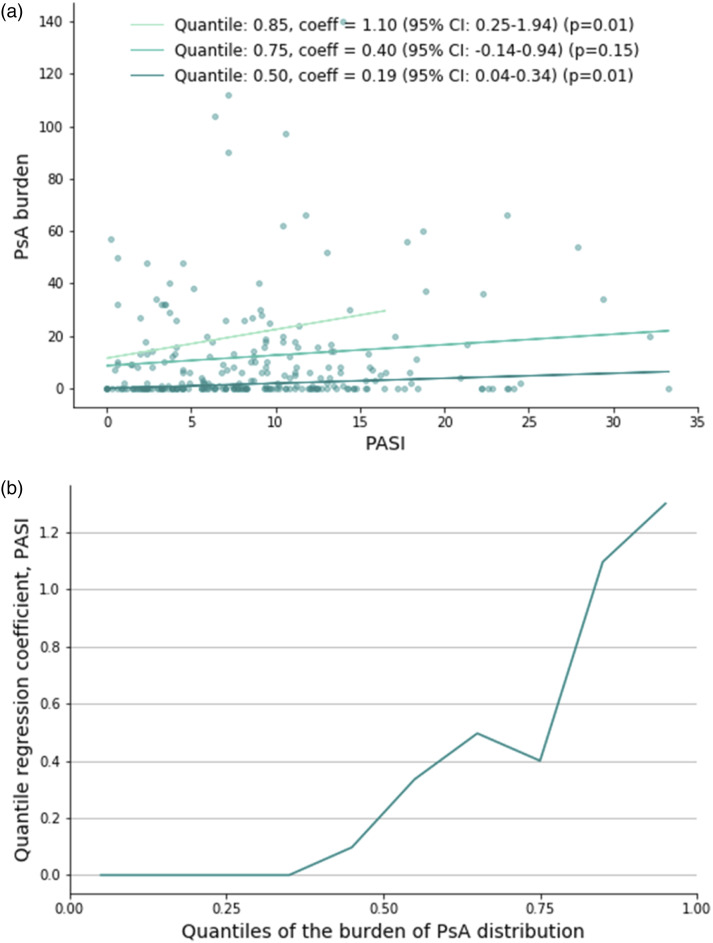
(A) Quantile regression lines for three different quantiles. (B) Regression coefficients as a function of the quantile. Baseline data (first treatment) is included for patients with a diagnosis of PsA.

The results of the quantile regression exploring the relationship between PASI and the burden/severity of PsA were plotted (Figure 4). The upper panel (a) visualizes three different regression lines for different quantiles (.5 (median), .75, and .85). The median (.5 quantile) regression yielded a coefficient of 1.10 (95% CI: .25-1.94), indicating that PASI had a significant positive effect on the median severity of PsA (*P* = .01). The coefficient obtained from the .75 quantile regression was .40 (95% CI: −.14-.94), indicating that PASI was not significantly associated with the .75 quantile of the burden of PsA. The .85 quantile regression resulted in a coefficient of .19 (95% CI: .04-.34), signifying that PASI had a significant effect on the .85 quantile of the burden of PsA as well. The lower panel (b) indicates that the relationship between PASI and the burden of PsA is stronger for higher values of PASI.

## Discussion

Our study investigated the predictability of patients with psoriasis developing PsA. We demonstrated that machine-learning models could differentiate between patients with and without PsA with an AUROC of 73.7% when considering the number of prior treatment series, and AUROC = 72.2% when not. Variables with the highest discriminatory power were general physical well-being (EQ-5D), PASI, and the presence of nail psoriasis. Moreover, our study highlighted that the model’s learned relationship between predictive variables and the target variable was often non-linear. This indicates that simpler models limited to linear functions may be insufficient in the more detailed descriptions of patterns among patients with psoriasis and PsA.

Importantly, the model that did not use information on previous treatment attempts classified significantly more first treatment series in the PsA group among patients who had no PsA diagnosis at baseline but developed PsA during treatment, compared to patients who already had PsA at baseline. Consequently, patients who are diagnosed with PsA in the future resembles patients who already have the diagnosis for this model, suggesting the potential for predicting PsA in patients with psoriasis at an earlier stage.

The detection of PsA at an earlier stage would be of clinical importance, since it could help dermatologists selecting the optimal therapy for simultaneously treating psoriasis and PsA. Some biologics are very effective in treating both diseases, and therefore, could be a good choice for patients with psoriasis in high risk of developing PsA.

Ideally, dermatologists can use their unique position to prevent permanent joint damage and deformation caused by severe PsA in some patients by facilitating early treatment and involvement of rheumatologists. This could potentially be an interesting future step towards personalized medicine.

Previous research on the SDNTT data shows that treatment goals and outcomes vary among individuals,^18,19^ but hopefully, detecting and treating PsA earlier will improve the burden of disease and treatment satisfaction for patients with psoriasis.

Previous studies^20,21^ showed a positive association between nail involvement of psoriasis and risk of developing PsA. This is in agreement with our results, where severity of nail psoriasis was found to be a predictor of PsA. Furthermore, one of the studies^20^ concluded that treatment with biologics decreased the incidence of PsA among patients with psoriasis. This highlights the importance of early detection of PsA facilitating earlier treatment with therapies appropriate for treating both diseases simultaneously.

Interestingly, both median PASI and BSA are higher among patients without PsA compared to patients with PsA. Conversely, physical well-being measured by EQ-5D is better among patients without PsA. Therefore, the models found a negative relationship between having PsA and PASI but a positive relationship between having PsA and physical impairment. We can speculate that if the life quality and physical well-being are severely impaired while the severity of psoriasis is more moderate, a large proportion of the disease burden might be attributed to symptoms of PsA. Additionally, previous treatment of psoriasis may have contributed to the improvement of the skin manifestations of psoriasis.

The quantile regression indicated that, although the median PASI was lower among patients with PsA compared to patients without PsA, there was a positive correlation between median PASI and severity of PsA among patients diagnosed with PsA. The positive association between severity of psoriasis and severity of PsA is consistent with previous research.^20,22^

### Limitations

This study was limited by the modest number of patients. Especially a larger number of patients with a PsA diagnosis registered after inclusion in the registry could provide essential insight into the development of PsA among patients with psoriasis. A larger number of incident cases would make estimates more accurate, especially if data at the time of PsA diagnosis were available.

Additionally, since data have been collected exclusively from patients treated in Switzerland, mainly in Zürich, our conclusions might not be generalizable to other countries. Further studies with more data from other geographical regions would be valuable to better elucidate the topic.

### Conclusion

In conclusion, machine-learning models successfully distinguished between patients with and without PsA. Factors such as PASI, physical well-being, age at diagnosis, age at treatment start, and severity of nail involvement of psoriasis were particularly important in the distinction. Notably, the model found that patients who were first registered with PsA after their inclusion in the registry, resembled patients who already had PsA at the time of inclusion. This indicates that a future diagnosis of PsA can be predicted to some extent for patients with psoriasis. These results may support the early detection of PsA, enabling timely and appropriate treatment and ensuring the necessary involvement of a rheumatologist.

## Supplemental Material

Supplemental Material - Predicting Psoriatic Arthritis in Psoriasis Patients – A Swiss Registry Study Supplemental Material for Predicting Psoriatic Arthritis in Psoriasis Patients – A Swiss Registry Study by Mia-Louise Nielsen, Troels C. Petersen, Lara Valeska Maul, Jacob P. Thyssen, Simon F. Thomsen, Jashin J. Wu, Alexander A. Navarini, Thomas Kündig, Nikhil Yawalkar, Christoph Schlapbach, Wolf-Henning Boehncke, Curdin Conrad, Antonio Cozzio, Raphael Micheroli, Lars Erik Kristensen, Alexander Egeberg, and Julia-Tatjana Maul in Journal of Psoriasis and Psoriatic Arthritis®
