# Supplementary material for: Predicting Psoriatic Arthritis in Psoriasis Patients – A Swiss Registry Study
Source: J Psoriasis Psoriatic Arthritis. 2023 Nov 21;9(2):41–50. doi: 10.1177/24755303231217492 (PMC11361495; doi:10.1177/24755303231217492)

Supplementary figure 1: SHAP values for the most important features for models 1: (a-c), and for model 2: (d-f). The SHAP values explain predictions/classifications by the model, i.e., the model classifying patients in the PsA group results in higher SHAP values.

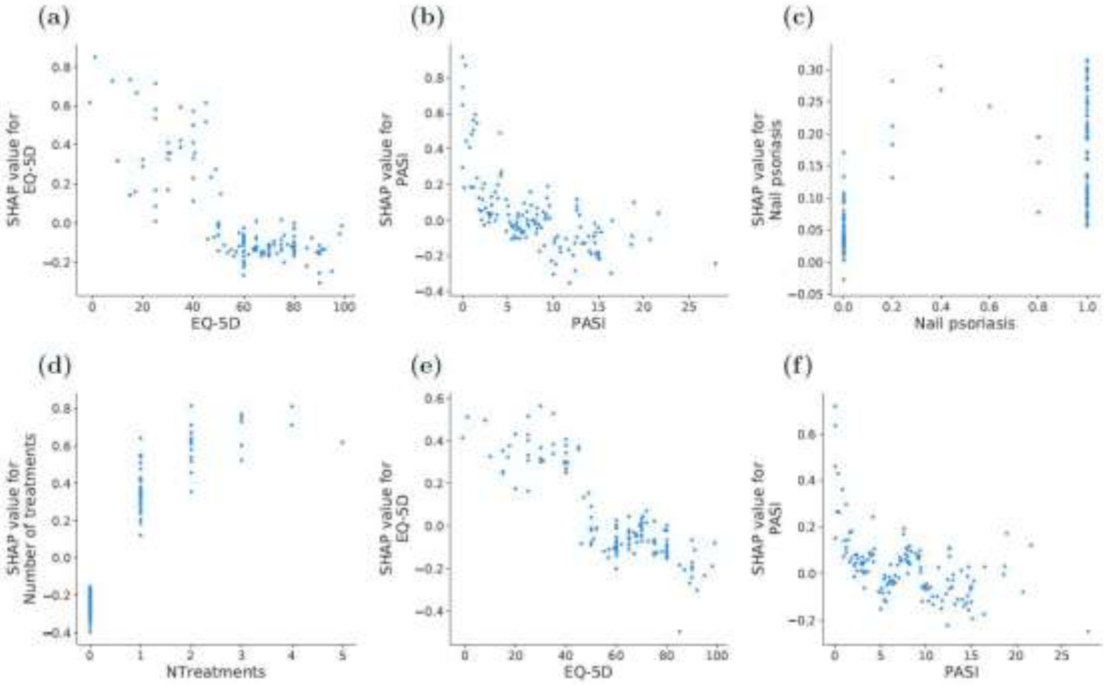

Supplement: Supplemental Material - Predicting Psoriatic Arthritis in Psoriasis Patients – A Swiss Registry Study [file sj-pdf-1-jps-10.1177_24755303231217492.pdf]
